# A Game to Deal With Alcohol Abuse (Jib): Development and Game Experience Evaluation

**DOI:** 10.2196/11151

**Published:** 2019-10-15

**Authors:** Darlinton Barbosa Feres Carvalho, Daniel Bueno Domingueti, Sandro Martins De Almeida Santos, Diego Roberto Colombo Dias

**Affiliations:** 1 Federal University of São João del-Rei Departament of Computer Science São João del-Rei Brazil; 2 Federal University of Amazonas Department of Anthropology Manaus Brazil

**Keywords:** alcohol abuse, serious game, software design, proof of concept evaluation

## Abstract

**Background:**

Alcohol abuse is the primary cause of (public) health problems in most parts of the world. However, it is undeniable that alcohol consumption is a practice that is widely accepted socially in many places, even being protected by law as a cultural and historical heritage. The issue of alcohol abuse is complex and urgent, and consequently, it is necessary to create innovative approaches such as the proposal explored in this study.

**Objective:**

This study aimed to explore the development and evaluation of a serious game for smartphones to present a novel approach to address the issue of alcohol abuse.

**Methods:**

A serious game was developed to instill the consequences of alcohol abuse into the player through experimentation in the game. In the game, the consequences of alcohol use are demonstrated by increasing the game speed that gives an illusion of fun but also leads to a premature death. The evaluation employed an assessment based on the Alcohol Use Disorders Identification Test (AUDIT) and the Game Experience Questionnaire (GEQ). The participants belonged to the university student’s house.

**Results:**

The game development process has been presented, including its mechanics and gameplay. The game has the style of action and adventure games in which the player controls an indigenous avatar that can deflect or attack opponents coming his or her way. The game evaluation comprised an assessment based on 23 participants, aged 20 to 29 years. According to the AUDIT assessment, 18 participants reported having a low or nonexistent degree of alcohol dependence and 5 declared average dependence. Regarding their habit of playing games on smartphones, 9 participants declared they have this habit of playing (habitual players), and among the 14 that did not have this habit of playing (nonhabitual players), 3 participants declared not having a smartphone at all. The GEQ core assessment showed a higher positive affect among the participants with a habit of playing games, scoring 2.80 (habitual players) on a scale of 4.0 versus 1.61 (nonhabitual players), and higher tension as an opposite relationship of 0.81 (nonhabitual players) versus 0.37 (habitual players). The overall GEQ evaluation showed that the game presents a more positive than negative affect on all users, besides showing the other desirable characteristics of serious games.

**Conclusions:**

We present a new way of dealing with the issue of alcohol abuse through a game designed for smartphones. It promotes an overall positive user experience, having a greater impact on users accustomed to games. The proposed approach has its niche, though it is still a minority in the evaluated population. Further research should explore new game features, such as new styles, to make the game more attractive to a wider audience, in addition to performing an in-depth study on the effects of playing it.

## Introduction

### Background

Alcohol abuse is the primary cause of (public) health problems in Brazil. According to the World Health Organization (WHO), in 2012, about 3.3 million deaths or 5.9% of all deaths recorded on the planet were attributable to alcohol consumption [1]. The abusive consumption of alcoholic beverages is the component cause of more than 200 types of diseases and injuries. Alcoholism represents harm to private health, public health, and the economy as a whole. The estimated annual cost of alcoholism is £20 billion in the United Kingdom and more than US $200 billion in the United States. Brazil loses around 7% of its gross domestic product per year because of excessive consumption of alcoholic beverages. The cost estimated in 2014 reached R$372 billion [2].

However, it is undeniable that alcohol consumption is a part of many cultures, being a practice that is widely accepted socially in most parts of the world. In Brazil, the spirit *cachaça* (a type of distilled spirit made from fermented sugarcane, the most popular distilled alcoholic beverage in Brazil) is even protected by law for its distinguished characteristics (Brazilian Presidential decree n.6871/2009) and is considered a part of national cultural and historical heritage. The issue of alcohol abuse is complex and urgent, and consequently, it is necessary to create innovative approaches such as the proposal explored in this study.

### Objectives

The main aim of this study was to explore the possibilities offered by new technologies to address this relevant issue. Numerous successful cases have been found in the literature employing serious games in dealing with relevant health care issues [3]. Considering the growing use of smartphones, the creation of a serious game for smartphones takes advantage of this important channel of communication to innovate an intervention for such issues.

Our proposal was to employ new technologies to raise awareness about the consequences of alcohol abuse, promoting empowerment of the individual, traditional cultures, and social responsibility. The authors believe that a suitable means for this is a playful educational tool, that is, a serious game. In this sense, the user is allowed to experience the option of drinking and modifying the gameplay, eventually increasing the fun factor, but also showing the consequences, such as the premature termination of the game (player’s death in the game).

## Methods

### Design Rationale

Game development is based on the premise that games are strongly linked to reality. According to Abt [4], a game has some core elements such as rules, participants, information, gains, and losses. It is also possible to assimilate factors such as competition, opposition, and maximization or minimization of some factor over another. Thus, it is feasible to relate a game to other human activities, as all have rules (of society or a particular environment), participants, successes, and failures (of tasks or procedures involved).

An indigenous hero is considered for the setting of the game. It is worth noting that alcohol abuse also extends in Brazil to the indigenous sphere of society. Langdon [5] points out that because of the process of inclusion of the indigenous people in the broader society, they have begun to substitute or add the distilled beverage into their everyday life. The consequences of this change have also been addressed by Viertler [6] and Guimarães and Grubits [7], demonstrating that alcohol abuse is the main cause of addiction diagnoses, accidents, and cases of violence against other members of the community, including the use of various weapons and incidence of fatal victims. The indigenous theme also brings out the problem in these societies to a wider audience as intervention in this area is also not an easy task and lacks attention in a broader sense.

As Ingold [8] points out, the learning process is about the education of attention. Thus, as a child, learning is consolidated through games and toys. Through them, the study by Ritterfeld et al explains [9], “children exploit and accumulate the world, improving their skills and abilities” because they are extremely motivating activities. However, later in elementary school, there is a clear separation between learning and fun, making learning somewhat unmotivating and unpleasant. This is because motivation dictates the learning flow—when motivation ends, learning and the act of playing also end [10].

Electronic games have been proposed to make learning motivational as they have the ability to communicate concepts and facts of many subjects effectively and allow people to recreate themselves in new worlds and achieve recreation and deep learning at the same time. That is, they are capable of creating a dramatic representation of the studied situation [4,10,11].

Such games are called educational games. However, they do not always achieve their goal as many prioritize the educational or entertainment components separately. A methodology is used specifically for the development of serious games that are games that use the artistic medium of the game to deliver a lesson or teach about some subject so that both components are balanced [9].

According to Michael and Chen [11], a serious game should contain some elements related to design and development itself, including the following: (1) simple (intuitive)—usable by people who have little or no experience with digital games, (2) adequate simulation—both in terms of realism and difficulty, and (3) progress analysis—it allows the player to formulate strategies, in case he or she learns what the game wants to pass to achieve greater progress in the game.

To achieve these desirable characteristics, the development was divided into 3 stages: (1) planning, (2) prototyping, and (3) app consolidation. The whole process was documented in the Game Development Document, which contains all the necessary information for game development, such as an execution flowchart, the definition of gameplay, and references and inspiration. This paper has presented a consolidated version of the Game Development Document, highlighting the main concepts employed in the game with regard to mechanics, gameplay, and implementation.

### Field Evaluation

The game evaluation was carried out with students from the student residence of the Federal University of São João del-Rei. This residence is the student housing provided by the university for low-income students under the university’s official assistance program. All participants were volunteers and had to sign a participant consent form, which described the general purpose of the study and stated the research procedures according to international and Brazilian ethical research laws and principles. The results are opinions declared by the participants and were disclosed anonymously. The proposed evaluation consisted of 3 stages: 2 questionnaires (pre- and postintervention) and a 20-min intervention phase of user interaction with the game. No further explanation about the game was provided, leaving the exploration process up to the participants. When the participants asked about a specific game element, the feedback was an incentive for them to explore and test the interface to figure out the answer.

The preintervention interview was performed to characterize the participant by collecting information such as age, sex, and their level of alcohol dependence. To identify the level of alcohol dependence of the users, the Alcohol Use Disorders Identification Test (AUDIT) was employed. The WHO recommends the use of AUDIT [12] as a simple, brief method of screening for excessive drinking. Its questionnaire contains 10 multiple-choice questions with 5 choices each. The assessment works as a sum of points acquired by each alternative—from 0 to 4—and the final result, that is, the sum of all the alternatives, determines the level of alcohol dependence of the interviewee. The risk degree of dependency, potential consequences, and intervention recommendation for the interviewee are classified according to 4 levels. The correspondence among scores and the meaning of their risk level is shown in Table 1.

The Game Experience Questionnaire (GEQ) [13] was used for the postintervention evaluation. It consists of a questionnaire composed of 3 modules: the core, the postgame, and the social modules. The questionnaire has simple and direct questions about the player’s thoughts and feelings, such as “I felt happy” and “I felt angry.” The core module has questions regarding feelings and beliefs that the interviewee had while playing. In the postgame module, the questions are focused on when the player stopped playing. In this way, the participant demarcates the level of agreement with the corresponding sentence on a scale from 0 to 4, where 0 means no agreement and 4 means a lot of agreement.

Only the core and postgame modules were used as the proposed game does not have social interactions. The participants answered them after the intervention stage. In each module, a group of questions defined a component to be analyzed, and the final calculation of it was given by the simple average of the questions. The total average of the components was given by the simple average of all the individual components of the interviewees—with results ranging from 0 to 4. In this case, 0 means that there is no presence of that component and 4 means there is a lot of it.

Finally, the postintervention evaluation consisted of 3 questions to evaluate the design of the app concerning the visual and sound resources and the available information about the game (such as tutorials and information about the project) and about the controls. A scale from 0 to 4 was also used at concordance levels, where 0 meant weak and 4 meant optimal.

**Table 1:** Risk level scores with regard to alcohol consumption behavior according to the Alcohol Use Disorders Identification Test assessment.

Score	Risk level
0-7	Inexistent: low-level drinking or abstinence
8-15	Low: alcohol use in excess of low-risk guidelines
16-19	Medium: harmful and hazardous drinking
Over 20	High: alcohol dependence

## Results

### Development

The Game Development Document details the proposed modeling and the definition of the rules, basic interface, and other details. The details of the graphics and sound features were being defined along with the game development. In total, 2 versions were developed. The first prototype was made in the Massachusetts Institute of Technology (MIT) AppInventor platform, but its development was discontinued because of performance issues. The last prototype is the evolution of the first one but using the Unity Graphic Engine (ie, Unity3D) for its implementation.

The game was deployed on the Android operating system because of its popularity. The development of an app through native language is the development of an Android app exclusively using the Android Software Development Kit or Android Native Development Kit. This allows for efficient development as the developer has direct access to all the tools of the device. Access to all tools allows you to design the app in the best possible way for the operating system, maximizing the user experience. However, this environment requires mastery of the platform’s native language and its tools. All physical modeling involved in the app must be developed. For easy game development, many platforms can be found in the Android ecosystem that assist developers in creating new games.

A study was carried out on game development platforms available for the Android system. The most promising ones were MIT AppInventor and Unity Graphic Engine.

The MIT AppInventor platform is an Android app development platform supported by Google and maintained by the MIT (United States). It has a drag-and-drop system where users select, drag, and fit blocks that determine a line or method of execution in an interaction area. It is an open-source license environment that is easy to learn, with the freedom to run the program for any legal purpose.

At first, the authors chose the MIT AppInventor platform for prototyping because of the small learning curve and the need to set implementation details. It was a straightforward implementation of what was designed. However, because of performance issues when the game had to perform fast updates of the graphic elements on the screen (ie, the game starts experiencing unexpected screen update errors), a more robust platform was needed. Therefore, the game’s final version was fully implemented using the Unity Graphic Engine. It took a while to learn this new platform, but the process of prototyping in AppInventor to achieve better implementation later in Unity was shown to be effective to enable fast prototyping, especially considering programmers without much experience in game development, and produce a better game in terms of quality on a more robust platform.

**Figure 1:**
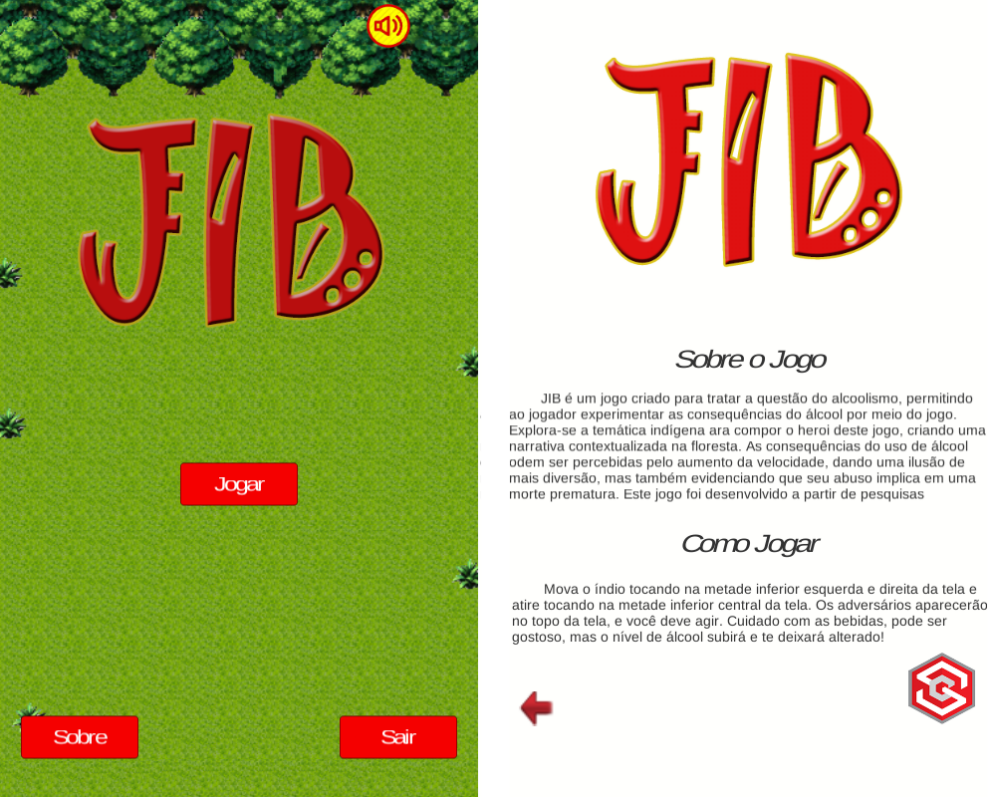
Two screens of the proposed game: (left) first screen; (right) about the game screen (in Portuguese).

The proposed game follows the style of action and adventure games, in which the player controls an indigenous avatar who can deflect or attack opponents coming his or her way. It has 3 screens: the initial menu screen, an information display screen about game rules and the project, and the screen that contains the game. The initial menu screen, described in Figure 1 (left), allows you to move to any other screen within the app and to exit the app. The about screen, shown in Figure 1 (right), allows the user to return to the initial menu screen (using the return button in the upper left corner). Information regarding the game screen (and the game itself) is described in the following subsections. It is important to note that all graphics and sounds are available on the internet under the Creative Commons license.

The instructions presented in the about screen (Figure 1 [right]) can be translated as follows: *About the Game* – “JIB is a game created to address the issue of alcoholism, allowing the player to experience the consequences of alcohol abuse through the game. The indigenous theme has been explored to compose the hero of this game, creating a narrative contextualized in the forest. The effects of using alcohol can be perceived by increasing the game speed and giving an illusion of more fun but also by showing that alcohol abuse might lead to premature death.”; and *How to Play* “Move the Indian by touching the lower half left and right of the screen and shoot by touching the center of the screen. Opponents will appear at the top of the screen, and you must act. Be careful with the drinks, it can be tasty, but the alcohol level will rise, and it will change you.”

**Figure 2:**
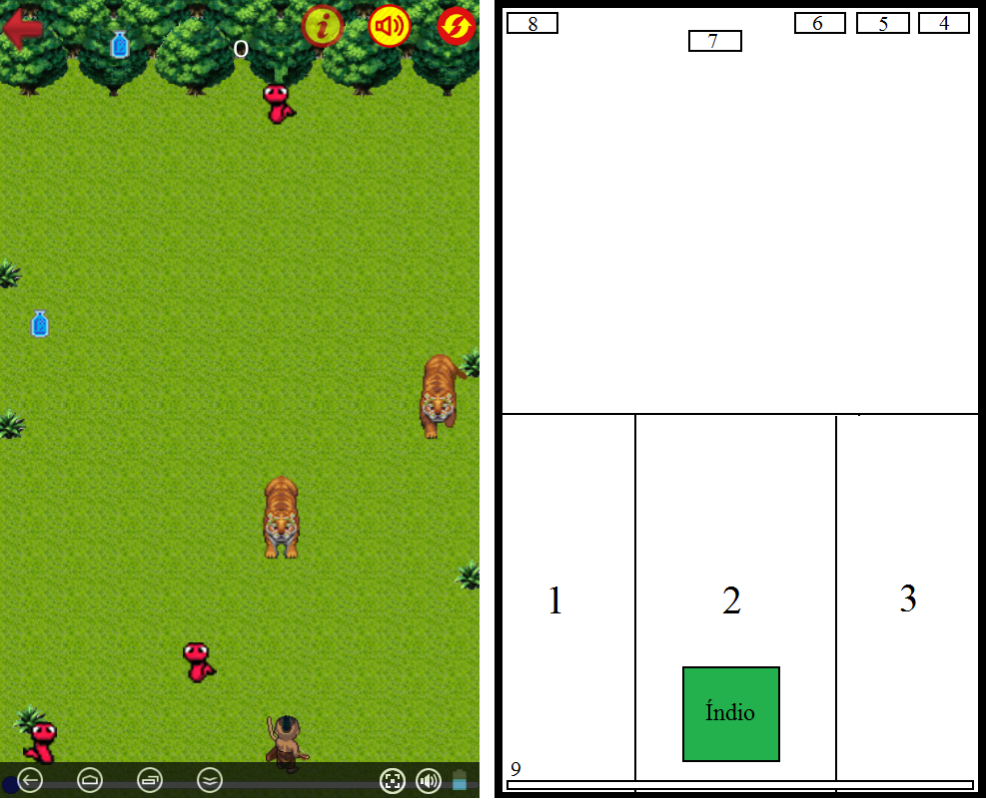
User interface with interaction elements positioning: (left) game play screen; (right) sensible areas of the interface for user interaction.

The user interaction consists of using a touch screen, where areas are defined for interaction and arrangement of the elements of the game. Figure 2 (right) presents a schematic of these areas in comparison with the game screen (Figure 2 [left]: (1) move character to the left, (2) shoot the arrow, (3) move character to the right, (4) restart the game, (5) enable or disable the sound in the game, (6) enable or disable the display of indicative signs of the action buttons, (7) display the player’s current score, (8) return to the home screen, and (9) indicate current alcohol consumption level).

The player, as already mentioned, controls an indigenous avatar who can deflect or shoot arrows at the opponents that appear in his or her way. Opponents appear on the upper edge of the device screen, and when they reach the lower end, they disappear. Some defined opponents are the snake, the jaguar, and the drink. Once the Indian comes in direct contact with the snake or the jaguar, the game ends. If the arrow hits an opponent, the player gets a score that is added and displayed on the screen.

When the player consumes the drink, it increases the points and speed of the game, which in a way also increases the fun. The idea is that drinking causes the player to slow down compared with the world, that is, the other elements get faster.

**Figure 3:**
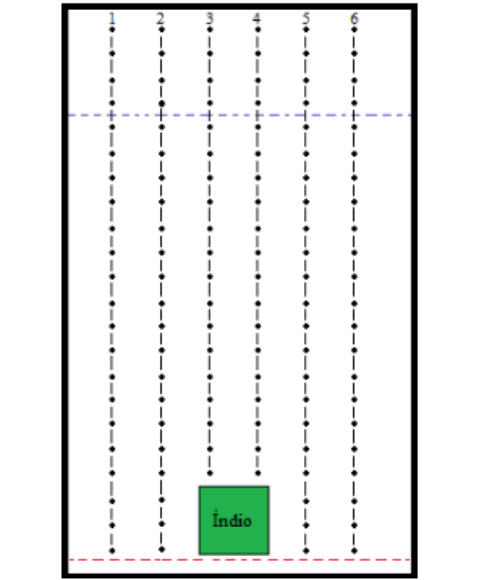
Enemy movement lanes in the game screen.

The opponents use a system for continuous displacement, explained in Figure 3, to avoid the overlap and improper collision of opponents on the screen. The lanes have counters associated with them to notify their availability (or not). At the launch of an opponent, the counter of the chosen and adjacent lane is incremented. Counters are decreased when an opponent passes a safety range (represented by the horizontal blue dashed line closer to the top in Figure 3).

### Evaluation

Data were collected from a total of 23 participants, 12 of whom were male and 11 were female. The participants’ ages ranged from 20 to 29 years, although 2 participants preferred not to declare their age. Regarding the degree of dependency identified by the AUDIT questionnaire [12], 5 claimed not having any alcoholic dependence, 13 reported having a low or nonexistent degree of dependence, and 5 declared an average dependence. No participant declared a high degree of dependency.

Of the 23 participants, only 3 did not have a smartphone. Among those who owned a smartphone, 11 did not use it for games.

### Game Experience Questionnaire

The GEQ [13] was used to gain an understanding of user experience with the game. Overall, 2 GEQ modules were used: core and postgame.

First, the elements of the questionnaire should be understood. According to Johnson et al [14], the components—competence, stress, negative affect, and positive affect—are self-explanatory. Negative affect and positive affect refer to the user experience. For example, a user may feel good or unmotivated about the game. Challenge aims to present data regarding the amount of effort, difficulty, and pressure felt by the user during the game. Tension is related to the player’s frustration while playing the game. Flow aims to identify how interested the user was during the game. Immersion shows how immersed the user was with the story and elements of the game. Finally, competence is related to the player’s ability and how well he or she performed during the game. All core elements of the GEQ are presented in Table 2.

The GEQ postgame questionnaire was used to identify how the player felt after playing. This questionnaire is composed of 4 variables: positive affect, negative affect, tiredness, and return to reality. Positive affect is related to the satisfaction, victory, and power of the user after playing. In contrast, the negative affect addresses the user’s bad experiences after playing. Tiredness is related to the user’s exhaustion during the game. Finally, the returning to reality component addresses the user’s disorientation after a gaming period. Table 3 presents the postgame elements and related questions.

**Table 2:** Core Game Experience Questionnaire components and response options.

Core Game Experience Questionnaire components	Response options
Immersion	I was interested in the game’s story It was aesthetically pleasing I felt imaginative I felt that I could explore things I found it impressive It felt like a rich experience
Flow	I was fully occupied with the game I forgot everything around me I lost track of time I was deeply concentrated on the game I lost connection with the outside world
Competence	I felt skillful I felt competent I was good at it I felt successful I enjoyed it
Negative affect	It gave me a bad mood I thought about other things I found it tiresome I felt bored
Tesnion	I felt annoyed I felt irritable I felt frustrated
Challenge	I thought it was hard I felt pressured I felt challenged I felt time pressure I had to put a lot of effort into it

**Table 3:** Postgame Game Experience Questionnaire components and response options.

Postgame Game Experience Questionnaire components	Response options
Positive affect	I felt revived It felt like a victory I felt energized I felt satisfied I felt powerful I felt proud
Negative affect	I felt bad I felt guilty I found it a waste of time I felt that I could have done more useful things I felt regret I felt ashamed
Tiredness	I felt exhausted I felt weary
Returning to reality	I found it hard to get back to reality I felt disoriented I had a sense that I had returned from a journey

### Analysis

Analyses of the results from the core and postgame GEQ questionnaire (Tables 2 and 3, respectively) are presented as follows. The mean score was calculated for each component in both questionnaires. The average score was calculated for 3 different classes of users: nonhabitual players, habitual players, and total. Table 4 presents the results of the core questionnaire for the 3 distinct classes.

As shown in Table 4, the highest score found among users who declared themselves to be nongamers was the positive affect component (nonhabitual players=1.61), followed by flow and competence components (nonhabitual players=1.54). However, although the positive affect component obtained a higher score, it was similar to the negative affect component (nonhabitual players=1.25). Tension component was the one with the lowest score (nonhabitual players=0.81). The scores of the components, competence (nonhabitual players=1.54), immersion (nonhabitual players=1.44), and challenge (nonhabitual players=1.33), were higher than the general mean (mean 1.25).

The highest score was of the positive affect component (habitual players=2.8), followed by competence (habitual players=2.18), immersion (habitual players=1.96), flow (habitual players=1.89), challenge (habitual players=1.16), negative affect (habitual players=0.69) and, finally, tension (habitual players=0.37). It should be noted that in this evaluated class, the positive affect component scored considerably higher than the negative affect component. The scores obtained in the components, tension, negative affect, and challenge, were below the arithmetic mean (mean 1.57).

A combination of the 2 classes (nonhabitual players and habitual players) was also performed. The positive affect component had the highest score (total=2.08), followed by the components, competence (total=1.79), flow (total=1.68), immersion (total=1.64), challenge (total=1.26), negative affect (total=1.03), and, finally, tension (total=0.64). In the combination of the 2 classes, it can be noted that the positive affect component had a relatively higher score than the negative affect component. Only the components, challenge, negative affect, and tension, reached scores below the mean (mean 1.44).

With regard to the results obtained through the GEQ postgame questionnaire for nonhabitual players, the positive affect component reached the highest score (nonhabitual players=0.75). Negative affect was lower than the positive affect (nonhabitual players=0.5). The lowest value found was related to the return to reality component (nonhabitual players=0.43). The tiredness component obtained a relatively high value in comparison with the other components (nonhabitual players=0.68). The results referring to the GEQ postgame questionnaire for the 3 distinct classes are presented in Table 5.

For the habitual players class, the component with the highest score was the positive affect (habitual players=1.17), followed by the components return to reality (habitual players=0.48), negative affect (habitual players=0.2) and, finally, tiredness (habitual players=0). Positive affect reached a score considerably higher than negative affect. The components below the arithmetic mean (mean 0.46) were negative affect and tiredness.

The following scores were obtained for the 2 combined classes (nonhabitual players and habitual players): positive affect (total=0.95), return to reality (total=0.47), tiredness (total=0.43), and negative affect (total=0.40). Only the positive affect component had a score above the arithmetic mean (mean 0.56). However, this score was responsible for an increase in the mean.

The users also evaluated the proposed game interface in 3 aspects: (1) defined controls for the game, (2) quality of the graphics and sound, and (3) game information about the project and how to play. The simple arithmetic mean of these aspects was calculated and is presented in Table 6.

**Table 4:** Core Game Experience Questionnaire average scores for the 3 classes.

Core Game Experience Questionnaire	Nonhabitual players, mean	Habitual players, mean	Total, mean
Negative affect	1.25	0.69	1.03
Positive affect	1.61	2.80	2.08
Challenge	1.33	1.16	1.26
Tension	0.81	0.37	0.64
Flow	1.54	1.89	1.68
Immersion	1.44	1.96	1.64
Competence	1.54	2.18	1.79

**Table 5:** Postgame Game Experience Questionnaire average scores for the 3 classes.

Core Game Experience Questionnaire	Nonhabitual players, mean	Habitual players, mean	Total, mean
Positive affect	0.75	1.17	0.95
Negative affect	0.50	0.20	0.40
Returning to reality	0.68	0.68	0.43
Tiredness	0.43	0.48	0.47

**Table 6:** The average of usability aspects based on evaluation.

Aspect	Values (mean)
Game control	2.86
Graphics and audio resources	3.09
Available information	2.05

An important feature of this experiment was the division of participants into those who are habitual players and those who are not (nonhabitual players). It should be taken into account that the analyses performed by *players* tend to produce less variation as these participants have a basis for comparison with previous experiences.

During analysis of the results, it was verified that the participants more accustomed to playing games did not feel challenged by the Jib game. This can be explained by the difficulty of the game as the game does not have an increasing level of difficulty and can be saturated very quickly, using few iterations. Another factor that potentially influenced the perceived level of challenge in the game was self-declared competence.

The score of the immersion component was significant, even though the game was simple. Participants used to play games reported a good experience in this component. The returning to reality component scored 0 among participants who are habitual players. The authors believe that the habit of playing more complex games had a great influence on this component as the sense of reality of these people may be less influenced by Jib, and they could easily discern their virtual experience from the real one (returning to reality). In such cases, serious games with low complexity can possibly generate more significant results in people who are nonhabitual players.

In total, 2 participants were identified as outliers (participant 11 and 22). Both of them had much higher stress levels (tension) than the other participants. This component possibly had an influence on the negative and positive affect components. Participant 22 had the highest negative affect score among all the participants. Participant 11, on the contrary, obtained a score for positive affect within the average, which was not expected, given his or her level of tension.

## Discussion

### Related Studies

Although there is a vast body of literature available regarding serious games applied to health care, this is a recent topic, and therefore, there are only a few pieces in the literature related to serious games that deal with alcohol abuse.

Gaibler et al [15] presented a study that addresses the effect of alcohol on safe driving activity, especially in the young age group (between 14 and 27years). The app, a serious game, is a racing game in the third person, where the intention is to reach the final goal safely, avoiding alcoholic beverages in the process. Our proposal shares the principle of alcohol consumption affecting the gameplay. In this game, the players are challenged by lowering their game vision according to the level of intoxication. In our proposal, varying the game speed offers a more dramatic consequence to the user experience, leading to a premature end of the game. Nonetheless, this paper is limited to a discussion of some preliminary and promising results.

Rodriguez et al [16] conducted a systematic review related to serious educational games aimed at the consumption of alcohol and other drugs by adolescents. The search for papers was carried out in research portals. According to the authors, 8 papers related to the consumption of alcohol, cannabis, tobacco, methamphetamine, ecstasy, and other drugs were found. In addition, 6 other papers addressed the use of other drugs. However, only one of the papers showed a decrease in the frequency of drug use. The authors highlight the need for further investigation and development of serious educational games, such as the one presented in this paper.

Boendermaker et al [17] applied gamification techniques similar to a cognitive bias modification of attention (CBM-A) training task to draw attention away from images of alcoholic beverages. The applied training task is called a visual probe task [18,19] that consists of the use of pairs of images, where one presents an important stimulus to the alcoholic beverage and another one a neutral stimulus (something nonalcoholic). The activity consisted of 4 sessions, with at least a 1-day difference between sessions, for 2 weeks. The study was aimed at undergraduate students (96 students, mean age 21.2 years). The authors identified the problems of excessive alcohol consumption of the candidates by using questionnaires. The authors concluded that the innovation proposed in their study was insufficient to the task of motivating adolescents in training when compared with conventional CBM-A training. They believe that one of the motivations for such an outcome is related to the expectation that a game should be fun, a feature that was not the focus of their study. In this study, the focus is on building a game that is fun and fosters educational drive, but no analysis regarding user motivation to play the game or gameplay consequences has been performed.

More recently, Boendermaker et al [20] developed and evaluated a serious game aimed at increasing behavioral control in adolescents and thereby helping them to improve control over their alcohol use. The studied compared the game training to a game placebo and a nongame training version in a randomized controlled trial. The study sample was 185 adolescents (mean age 14.9 years) assessed for 4 weeks. As a result, the game variants were shown to motivate adolescents beyond the level of the nongame version. The exercised skill, behavioral control, improved significantly over time, but this effect was also present in the game placebo. Yet, as baseline drinking levels were low, no significant training effects on drinking behavior were found. Our study also employs a sample that has been characterized by having a low baseline on drinking. The authors believe the game activities alone may have had a beneficial impact on the measures of behavioral control.

Although the current results are not yet conclusive as to whether their game is effective regarding its purpose, the study shows exciting findings of the suitability of serious games. Serious games seem to be suitable to exercise new skills, but the evaluation of its effectiveness is still a major challenge. Nevertheless, their results, and our results, suggest that this is a promising way, and further research is required to disclose and assess its potential.

### Conclusions

The paper presented the development of a serious game for smartphones aimed at addressing the issue of alcohol abuse. The main purpose was to call the player’s attention to the consequences of alcohol abuse through experimentation in the game. The authors do not have the illusion that people would cease drinking immediately but do intend to contribute to the awareness of this social problem.

To evaluate the proposed approach, an assessment based on AUDIT and GEQ was carried out with residents of a student’s house. The proposed evaluation consisted of 3 stages: 2 questionnaires (pre- and postintervention) and 1 intervention stage, which consisted of 20 min of exposure to the game. The users were divided into groups based on their habit of gaming in the data analysis.

The quantitative analysis presents a high degree of positive affect concerning the participants who declared themselves to have the habit of playing, although they did not have a high level of challenge because of the low difficulty and small learning curve. Overall (among all participants), the positive affect score was twice that of the negative affect. In this way, the authors believe that the Jib game obtained a proper evaluation in the 2 different groups of participants, considering the limitations of this first version of the game.

In the future, the authors expect to continue to develop Jib and to increase the level of difficulty and application of the game. New experiments should also be conducted using different groups of users, such as indigenous people in urban contexts, and exploring the variations between gender, age, and family background related to the issue of alcohol abuse. The net effect of the gaming experience, with the implied alcohol use and fun, needs to be better studied and its suitability verified especially regarding addicts. Further research should also explore new game features, such as new styles, to make it more attractive to a wider audience, in addition to performing an in-depth study on the effects of playing the game.

## Abbreviations

AUDIT: Alcohol Use Disorders Identification Test
CBM-A: cognitive bias modification of attention
GEQ: Game Experience Questionnaire
WHO: World Health Organization
